# The WELL diet score correlates with the alternative healthy eating index‐2010

**DOI:** 10.1002/fsn3.1558

**Published:** 2020-05-06

**Authors:** Sparkle Springfield, Kristen Cunanan, Catherine Heaney, Katy Peng, Christopher Gardner

**Affiliations:** ^1^ Stanford Prevention Research Center School of Medicine Stanford University Palo Alto CA USA; ^2^ School of Medicine Stanford University Palo Alto CA USA

**Keywords:** AHEI‐2010, diet quality, FFQ, survey measures, WELL

## Abstract

The quality of one's overall diet has proven to be of great importance to health and well‐being. Unfortunately, diet quality is time‐consuming to assess. The Stanford Wellness Living Laboratory (WELL) administered an online survey that included the WELL Diet Score (a novel diet quality assessment calculated from 12 diet‐related items). Subsequently, WELL participants were asked to complete the 127‐item Block Food Frequency Questionnaire (FFQ) online. The present study's primary objective was to compare the WELL Diet Score with the established FFQ‐based Alternative Healthy Eating Index‐2010 (AHEI‐2010), in a subset of WELL participants (*n* = 248) who completed both dietary measures through WELL’s online platform. The two scores were significantly correlated (*r* = .69; *p* < .0001). Regression analyses demonstrated that the WELL Diet Score was positively significantly associated with sociodemographic determinants of diet quality and protective health factors, including older age, higher education, lower BMI, and higher physical activity. In summary, the WELL Diet Score, derived from 12 small diet‐related items that can be completed in 5 min, was significantly positively correlated with the AHEI‐2010 derived from the lengthy 127‐item FFQ, suggesting the potential utility of the WELL Diet Score in future large‐scale studies, including future WELL studies.

## Graphical abstract

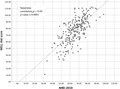

## 1 INTRODUCTION

While the quality of one's overall diet is linked to chronic disease prevention and well‐being, it is time‐consuming to assess. Nutritional epidemiologic studies tend to quantify hypothesis‐driven diet quality using a priori—derived indices that measure adherence to established, evidence‐based dietary patterns for chronic disease prevention (Alkerwi, 2014; Chiuve et al., 2012; Hu, 2002; McCullough et al., 2002; Reedy et al., 2014; Schwingshackl & Hoffmann, 2015). In recent years, several of these diet quality indices have been created and associated with lower risk of chronic disease and all‐cause mortality (McCullough et al., 2002). For example, the Alternative Healthy Eating Index‐2010 (AHEI‐2010) is recognized as a leading method and widely used for predicting diet‐related chronic disease outcomes (Chiuve et al., 2012; McCullough et al., 2002). Nevertheless, there is not a “gold‐standard” index or consensus as to the definition of diet quality (Alkerwi, 2014).

Diet assessments used in observational studies typically include 24‐hr diet recalls and food frequency questionnaires (FFQ). Diet quality index scores can be derived from 24‐hr diet recall and FFQ data. However, both methods tend to be time‐consuming and costly, especially when research staff are involved in data collection (Shaghaghi, Bhopal, & Sheikh, 2011). For example, the 24‐hr diet recall requires reporting an entire day of self‐reported dietary intake (Young & Nestle, 1995). While collections of multiple 24‐hr diet recalls are superior to a single day of diet data, each additional recall adds burden to participants. Evidence suggests that online, self‐administered 24‐hr dietary recalls are useful in large studies, albeit they may not be well received among older participants or certain population groups (Ettienne‐Gittens et al., 2013; Frankenfeld et al., 2012). Alternatively, a single self‐administered food frequency questionnaire (FFQ) can be relatively more efficient than multiple 24‐hr diet recalls, because it probes participants for estimates of typical dietary intake over specified time ranges (in windows of six months to one year), using a list of ~110–150 food items and food groups, and does not require an interviewer (Steinemann et al., 2017). However, FFQs typically require 30–60 min to complete, which can be a burden for participants, especially when it is administered as part of larger set of questionnaires (Steinemann et al., 2017). Given these considerations, it is desirable to have tools that can assess diet quality with reasonable accuracy in a short amount of time (≤5 min). Such an index could be used in future observational research but may also have potential application in the clinical setting.

To this end, our research team developed a short self‐administered online survey that aims to measure diet quality. The primary objective of the present study was to compare the WELL Diet Score (calculated from 12 short diet‐related items embedded in the WELL survey) with the established Alternative Healthy Eating Index‐2010 (AHEI‐2010), derived from the 127‐item Block Food Frequency Questionnaire (FFQ). It is important to note that the AHEI‐2010 is not considered the gold‐standard diet quality assessment tool; thus, the present study is not seeking to use it to validate the WELL Diet Score. Instead, we seek to test the rigor of our original WELL Diet Score against the established AHEI‐2010 to support its use in the WELL study and potentially in future studies.

## 2 MATERIALS AND METHODS

### 2.1 Study Design

As an initiative developed in the Stanford Prevention Research Center (SPRC), investigators are using WELL to generate comprehensive scientific data to help define, understand, and improve well‐being among people from diverse backgrounds. Based on emergent themes from one hundred semi‐structured qualitative interviews, the WELL survey is a 76‐item instrument, focused on 10 domains of well‐being “paper under review.” As of June 2019, 4,248 women and men, 18 years or older, have completed the survey. Details about the WELL study design, protocol, informed consent measures, and recruitment are available elsewhere “paper under review.”

The present study is a cross‐sectional analysis on 248 WELL study participants who completed both a WELL online survey that included 12 diet‐related questions and a Block FFQ up to one year apart. Completion of the FFQ was optional. Up to four email reminders were sent to encouraged participants to fill out the Block FFQ (Guy et al., 2012; Houston et al., 2010; McLean et al., 2014).

### 2.2 Dietary assessments

#### 2.2.1 WELL diet survey

The WELL diet survey elicited information about the frequency of dietary intake and meal preparation behaviors. Participants were asked how frequently they consume the following diet‐related items: (a) vegetables, (b) fruits, (c) whole grains, (d) beans or lentils, (e) sugar‐sweetened beverages (including 100% fruit juice), (f) red/processed meats, (g) nuts and seeds, (h) high‐sodium processed foods, (i) sugar‐sweetened baked goods or candy, and (j) fish. They were also asked how frequently they engaged in the following behaviors: (h) preparing meals at home and (i) eating fast food (e.g., McDonald's). These items were included based on the expert opinion of SPRC nutrition professionals, and evidence suggesting preparing and consuming foods at home is positively associated with diet quality (Hartmann, Dohle, & Siegrist, 2013; Todd et al., 2010; Wolfson & Bleich, 2015; Mancino, Todd, & Lin, 2009).

To minimize measurement error and to reduce participant cognitive burden, responses to these questions regarding frequency of consumption used a branching technique (Malhotra, Krosnick, & Thomas, 2009). First, participants reported if they consumed the food (or engaged in the behavior) less than once a week, every week but not every day, or every day. Depending on their response, participants were then offered a set of more specific responses. For example, participants who reported “less than once a week” were then provided the following choices: never, 1 time in the past month, and 2–3 times in the past month. Participants who first reported “every week but not every day” were provided the more specific choices of 1–2 times a week, 3–4 times a week, and 5–6 times a week. Lastly, those participants who initially reported “every day” were asked how often in a day: 1 time a day, 2–3 times a day, 4–5 times a day, or 6 or more times a day. This strategy created 10 mutually exclusive ordinal responses with participants only being presented with 3 or 4 choices at a time.

For the 12 diet‐related items, the team of nutrition professionals working on the project agreed, by consensus, how to distribute points across the different frequencies of consumption. There were 10 possible frequency levels: Never, 1/month, 2‐3/months, 1‐2/weeks, 3‐4/weeks, 5‐6/weeks, 1/day, 2–3/days, 4–5/days, 5–6/days. Notably, points were not distributed as simply one additional point for each incremental frequency, and points were not similarly distributed for each food category. For example, for “vegetables,” 0 points were assigned for both the categories of “Never” and “1/month,” and 10 points were assigned for both the categories of “4–5/days” and “5–6/days” suggesting the opinion of the group of nutrition professionals that a frequency of “1/month” was no better than “Never,” and that no additional health benefit was likely from going beyond 4–5/day. In contrast, for “nuts, seeds, and nut butters,” 0 points were assigned for “Never,” 1 point was assigned for 1/month, 10 points were assigned for “1/day” and “2–3/days,” and then decreasing points were assigned for frequencies greater than 3/days due to the opinion that intakes higher than “2–3/days” could be problematic in terms of excessive energy intake. The detailed scoring approach is contained in the supplemental information, see Appendix S1.

Scores were then combined to generate a total WELL diet quality score, ranging from 0 to 120. The estimated time to complete the 12 diet‐related items is approximately 4 min based on our survey analytics.

#### 2.2.2 Block food frequency questionnaire

The Block FFQ is regarded as a leading instrument for diet assessment (Subar et al., 2001). It was derived from a food list gathered during two waves of National Health and Nutrition Examination Survey (NHANES) dietary recall data, 2007–2008 and 2009–2010. The reliability and validity of the 127‐item FFQ were established across a wide range of age, gender, income, and groups (Boucher et al., 2006; Norris et al., 1997; Steinemann et al., 2017; Subar et al., 2001).

WELL participants who completed a computer‐administered FFQ were asked to report on the frequencies and amounts of 127 different food and beverage items, in addition to completing questions to adjust for intake of fat, protein, carbohydrate, sugar, and whole grains. Of the 4,297 WELL participants, 299 opted to complete the FFQ. An early version of the WELL survey only included 5 out of the 12 dietary domains currently used in the WELL Diet Score; thus, the WELL Diet Score calculated for the first 50 participants was incompatible with the finalized scoring used here. We proposed, in advance, to exclude participants with an incomplete WELL diet survey and implausible total energy intakes (<500 kcal/day (*n* = 1) or >6,000 (*n* = 0) kcal/day), leaving a final sample of 248 for analysis. The total and component scores of the diet quality index AHEI‐2010 were calculated from the FFQ using Nutrition Quest (Berkeley, CA).

#### 2.2.3 Alternative healthy eating index

The AHEI‐2010 measures adherence to the Harvard Healthy Eating Plate through 11 dietary components that total 110 points. These include 6 adequacy‐focused components, such as servings of vegetables, fruits, whole grains, nuts and legumes, intake of fatty acids from fish, and intake of polyunsaturated fatty acids. There are also four avoidance components—including red meats, trans‐fats, sugary beverages, and sodium. Finally, there is one moderation component for alcohol consumption. For each component, scores range from 0 to 10 points (Chiuve et al., 2012). Similar to the WELL Diet Score index, a higher score means a better diet quality.

The WELL Diet Score (maximum total score 120) and AHEI‐2010 (maximum total score 110) consist of 12 and 11 individual diet components, each scored from 0 to 10, respectively. As illustrated in Table 1, these individual diet component scores have varying degrees of overlap, ranging from complete to none. WELL Diet Score and AHEI‐2010 individual component scores with complete overlap include vegetables, fruit, whole grains, sugar‐sweetened beverages, red meat/processed meat, and sodium. The individual components with little to no overlap include sugar‐sweetened baked goods or candy, fast food, and prepare your meal (cook food) for the WELL Diet Score, and trans‐fat and alcohol for AHEI‐2010. The remaining components: beans or lentils; nuts, seeds, or nut butter; and fish, have partial overlap.

**Table 1:** Comparison of the WELL diet score to the alternative healthy eating index 2010 (AHEI‐2010)

Individual components of the WELL Diet Score	Criteria for max WELL Diet Score (10)	Individual components of the AHEI‐2010	Criteria for max AHEI‐2010 (10)	Comparative description
Complete overlap				
Vegetables	≥4× day	Vegetables	≥5 servings/day	Both components focus on a variety on nonstarchy vegetables such as dark leafy greens
Fruit	≥2× day	Fruit	≥4 servings/day	Both components focus on whole fruits and exclude 100% fruit juices
Whole grains whole grain products	2–5× day	Whole grains	75–90 g/day	Both components focus on whole grains and foods featuring whole grains
Sugar‐sweetened beverages or 100% fruit juice	0	Sugar‐sweetened beverages or fruit juice	0 servings/day	Both components focus on sugar‐sweetened beverages
Red meat/processed meat	≤1× month	Red/processed meat	0 servings/day	Both component scores focus on red/processed meat
High‐sodium processed food	0	Sodium	Lowest decile mg/day	Both focus on sodium which is commonly found in processed foods
Partial overlap				
Beans or lentils	≥2× day	Nuts and legumes	≥1 servings/day	Both components contain beans and lentils, AHEI‐2010 focuses all legumes and nuts
Nuts, seeds, or nut butter	1–3× day	Polyunsaturated fatty acids (PUFA)	≥10% of energy	Both components contain polyunsaturated fatty acids (PUFA), WELL Diet Score focuses on nuts, seeds, or nut butter
Fish	≥1× week	Long‐chain (*n*−3) fats (EPA + DHA)	250 mg/day	Both components contain long‐chain (*n*−3) fats (EPA + DHA), WELL Diet Score focuses on fish
Little to no overlap				
Fast food	0	—	—	WELL Diet Score focused on fast food, AHEI‐2010 did not
Sugar‐sweetened baked goods or candy	0	—	—	WELL Diet Score focused on sugar‐sweetened goods, AHEI‐2010 did not
Prepare your own meal (cook food)	≥2× day	—	—	WELL Diet Score focused preparing your own meals, AHEI‐2010 did not
—	—	Trans‐fat	≥0.5% of energy	AHEI‐2010 focused on trans‐fat, WELL Diet Score did not
—	—	Alcohol	—	—

#### 2.2.4 Other measurements

For their potential associations with diet quality, the following sociodemographic characteristics were included in the univariate linear regression analyses: age, race/ethnicity, years of education, marital status, and work status. Other potential diet‐related health factors included self‐reported height and weight (used to calculate BMI), being a current smoker (i.e., current smoker versus nonsmoker), and physical activity (engaged in moderate physical activity (i.e., brisk walking) for 30 min or more at least 5 times per week versus not).

### 2.3 Statistical analysis

In addition to the WELL Diet Score and AHEI‐2010 total and component scores, standard descriptive statistics, including medians and inter‐quartile ranges, were used to describe participant characteristics and differences in the sociodemographic and health factors between FFQ completers and noncompleters. The total WELL Diet Score and the AHEI‐2010 were normally distributed; however, the individual component scores were not. See Appendix S2. Both Pearson and Spearman correlations were calculated and presented very similar results. Due to some of the scoring components having non‐normal distributions, Spearman correlations are reported for results. No adjustments were made for multiple testing due to the descriptive and exploratory nature of the study.

Additionally, univariate linear regression analyses were used to examine the association between the WELL Diet Score and sociodemographic determinants of diet quality and diet‐related health factors, including age, education, BMI, being a current smoker and physically active. See Appendix S2. A 5% level of significance was used, and all tests were two‐sided. To complete the analysis, the investigators used R (version 3.5.3), and IBM SPSS Statistics for Macintosh, Version 25.0 (Chicago, IL).

## 3 RESULTS

On average, WELL participants were predominantly female, white, young, middle‐aged (aged < 50 yr old), college educated, married, employed, had a normal (or slightly overweight) BMI, and were nonsmokers, see Table 2. Most achieved the recommended amount of physical activity but had room for improvement in diet quality, see Table 2. Compared with FFQ noncompleters, those who completed the FFQ were more likely to be older white women and more highly educated (e.g., postgrad and professional degrees), married, more physically active, and had moderately better diets as measured by our WELL Diet Score.

**Table 2:** Comparison of participant characteristics between WELL participants who completed the 12‐item WELL Diet survey + 127‐item FFQ (FFQ completers) and those who only completed the WELL Diet survey (FFQ noncompleters)

(a) Characteristics	Participants who completed the WELL Diet survey (*n* = 4,297)	
	FFQ completers (*n* = 248)	FFQ noncompleters (*n* = 3,999)
^a^Gender		
Female	189 (76.2)	2,791 (70.2)
Male	57 (23.0)	1,135 (28.6)
Transgender/fluid	2 (0.8)	49 (1.2)
Age categories		
18–35	77 ( 31.0)	1922 (48.1)
36–52	64 (25.8)	991 (24.8)
53–63	56 (22.6)	615 (15.4)
64+	51 (20.6)	471 (11.8)
Race/ethnicity		
White/Caucasian (including Hispanic origin)	191 (77.0)	2,577 (64.4)
Asian	46 (18.5)	982 (24.6)
Black/African American	7 (2.8)	213 (5.3)
Native American/Pacific Islander	3 (1.2)	208 (5.2)
Multiracial/another race	14 (5.6)	220 (5.5)
^b^Level of education		
High school or less	8 (3.0)	266 (7.5)
Some college	43 (19.4)	725 (20.4)
4‐year college degree	83 (37.4)	1,235 (34.7)
Postgrad/Professional degree	88 (35.5)	1,331 (37.4)
^c^Marital status		
Married or living as married (%Y)	158 (63.7)	2071 (52.0)
^d^Employment status		
Employed (% Y)	165 (66.8)	2,609 (65.6)
(b) Diet‐related health factors, *N* (%) and/or Mean (Std)		
^e^BMI categories		
<18.5 (Underweight)	8 (3.5)	123 (3.6)
18.5–24.9 (Normal)	119 (48.0)	1,870 (55.3)
25–29.9 (Overweight)	68 (27.4)	854 (25.3)
30.0 (Obese)	35 (14.1)	535 (15.8)
^f^Current smoker (% Y)	5 (2.0)	123 (3.1)
^g^Physical activity		
Engaged in moderate physical activity (i.e., brisk walking) for 30 min or more at least 5 times per week (% years)	140 (57.1)	1916 (47.9)
^h^WELL Diet Score, max 120	77.0 (18.1)	71.0 (18.9)

^a^ Gender, missing *n* = 0 (FFQ completers); missing *n* = 24 (FFQ noncompleters).

^b^ Level of Education, missing *n* = 26 (FFQ completers); missing *n* = 443 (FFQ noncompleters).

^c^ Marital status, missing *n* = 0 (FFQ completers); missing *n* = 22 (FFQ noncompleters).

^d^ Employment status, missing *n* = 1 (FFQ completers); missing *n* = 24 (FFQ noncompleters).

^e^ BMI categories, missing *n* = 18(FFQ completers); missing *n* = 617(FFQ noncompleters).

^f^ Current smoker, missing *n* = 0 (FFQ completers); missing *n* = 21(FFQ noncompleters).

^g^ Physical activity, missing *n* = 3(FFQ completers); missing *n* = 81 (FFQ noncompleters).

^h^ WELL Diet Score, missing *n* = 0 (FFQ completers); missing *n* = 37 (FFQ noncompleters)

**Figure 1:**
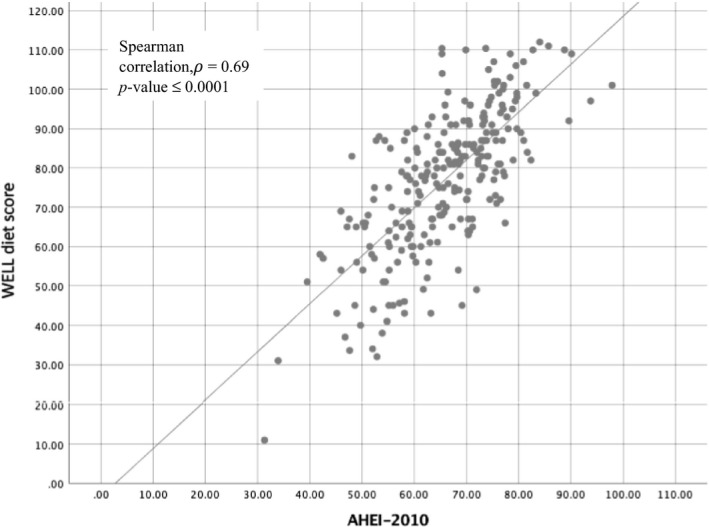
Simple scatter with fit line of the WELL diet score by AHEI‐2010 (*N* = 248)

Table 3 shows descriptive statistics (i.e., median; Q1, Q3) for the total scores and individual components of the WELL Diet Survey and AHEI‐2010. Using the standardized values, Spearman pairwise correlations are presented in Table 3 for the total scores and individual component. The median total WELL Diet Score was 79.0 out of a maximum of 120 (Q1: 65.0, Q3: 89.0), and the AHEI‐2010 was 66.5 out of a maximum of 110 (Q1: 58.7, Q3: 75.5). There is a significant, positive correlation between the two scores ( ρ=0.69;p0.0001 , see Figure 1.

**Table 3:** Description and correlation between the WELL Diet Score and the AHEI‐2010 total and individual component scores: medians (Q1, Q3) and Spearman pairwise correlations (*N* = 248)

WELL Diet Score, total and individual component scores (max)	Median (Q1, Q3)	AHEI‐2010, total and individual component scores (max)	Median (Q1, Q3)	Correlation ( ρ ) between AHEI‐2010 and WELL Diet Scores
Total score (120)	79.0 (65.0, 89.0)	Total score, (110)	66.5 (58.7, 75.5)	0.69***
Vegetables, (10)	9.0 (8.0, 9.0)	Vegetables, servings/day, (10)	3.9 (2.3, 5.9)	0.47***
Fruit, (10)	8.0 (4.0, 10.0)	Fruit, servings, g/days, (10)	4.5 (2.0, 7.9)	0.64***
Whole grain, 10)	4.0 (2.0, 8.0)	Whole grains, g/days, (10)	2.0 (1.0, 3.3)	0.51***
Sugar‐sweetened beverages or 100% fruit juice, (10)	8.0 (6.0, 10.0)	Sugar‐sweetened beverages and fruit juice, servings/day, (10)	9.0 (5.8, 9.7)	0.47***
Red meat or processed meat, (10)	6.0 (4.0, 8.5)	Red/processed meat, servings/day, (10)	7.5 (5.31, 8.8)	0.55***
High‐sodium processed foods, (10)	8.0 (4.0, 8.0)	Sodium, mg/days, (10)	6.4 (2.9, 8.9)	0.20***

*** *p* ≤ .001.

The individual diet components of the WELL Diet Score and AHEI‐2010 that focused on vegetables, fruit, whole grains, sugar‐sweetened beverages, red/processed meat, and sodium were correlated significantly, as expected since they were intended to measure the same behaviors/food choices. With the exception of sodium, all correlations were above 0.45 and thus considered satisfactory (Willett & Lenart, 2013). Additionally, univariate linear regression analyses revealed that a higher WELL Diet Score was significantly associated with older age, higher education, lower BMI, and higher physical activity, see Appendix S3.

## 4 DISCUSSION

Dietary assessment measures that are efficient, user friendly, and streamlined are essential to gather nutritional information and develop strategies to improve diets for chronic disease prevention. In this study, the WELL Diet Score derived from 12 diet‐related questions was positively and significantly correlated with the AHEI‐2010. Similar individual subcomponents in the two diet scores (e.g., vegetables, red/processed meats) were also significantly correlated. Furthermore, the WELL Diet Score demonstrated a significant association with established sociodemographic and health determinants of diet quality.

Consistent with our findings, other observational studies have highlighted the effectiveness of shortened versions of diet quality assessments in predicting diet‐related health outcomes (Funtikova et al., 2012; Schröder et al., 2011; Whitton et al., 2018). Overall, these findings provide further evidence that a diet quality score from a shortened dietary assessment tool can generate similar rankings of diet quality within a study population compared with those derived from a longer FFQ. Lower participant burden for diet assessment tools can allow for broader implementation and thus may be useful for understanding diet–disease relationships in populations of women and men from diverse sociodemographic backgrounds.

The study design and implementation involved several strengths. The WELL diet questions (12) were relatively easy to answer, as they are based solely on frequency—not portion sizes, which can be difficult to recall (Ervin & Smiciklas‐Wright, 2001; Harnack et al., 2004). Our study has the further advantage of enabling researchers to independently obtain a rapid assessment of diet quality online, without having to rely on a third party to calculate the diet quality index score, as can be the case with the AHEI.

Another strength involved the context in which the diet data were collected. Serving as both an observational and intervention study, WELL’s design can be used as an example for future studies. In addition to the 12 dietary items embedded in the lifestyle domain, the WELL survey collects information on 10 other domains of wellness and well‐being. These include social connectedness, stress and resilience, physical health, purpose and meaning in life, sense of self, financial issues, spirituality and religiosity, and exploration and creativity. This information can then be used to contextualize diet quality findings as well as inform the development of tailored and effective dietary interventions for residents of California's Northern Bay Area. In the future, the WELL study aims to extend the current work globally. As the study progresses, we intend to evaluate the WELL diet questions and corresponding WELL Diet Score in different sociodemographic, cultural, and environmental contexts.

While our survey offered a practical diet quality assessment for the WELL study, it had several limitations. First, although each participant who completed the WELL survey had up to 1 year to submit the FFQ, most (94%) opted to not complete a Block FFQ, underscoring the importance of minimizing participant's burden. Second, the variable lag time between the completion of the WELL Diet survey and the Block FFQ may have attenuated the correlation between the WELL Diet Score and the AHEI‐2010; the true correlation between the two metrics may be even stronger than observed and reported here. Third, both the WELL Diet Score and the AHEI‐2010 measures have unique limitations. The WELL Diet Score is based solely on the frequency of these behaviors; it does not estimate portion size, thereby lacking information on intake of total calories and micro‐ and macro‐nutrients. The FFQ, as a dietary assessment tool, is known to elicit high rates of under‐reporting (Kristal, Peters, & Potter, 2005). Fourth, reporting bias may have impacted both the WELL Diet survey and the 127‐item FFQ and corresponding diet quality scores, particularly in vulnerable populations, such as those who are overweight and obese, due to under‐reporting consumption of foods that have an unhealthy stigma (Alcantara et al., 2015; Klesges, Eck, & Ray, 1995; Krebs‐Smith et al., 2000; Tooze et al., 2007). Nonetheless, the 127‐item Block FFQ remains among the most accurate FFQs for assessing diet quality (Subar et al., 2001).

Fifth, there is also the potential for selection bias (Odgaard‐Jensen et al., 2011). Northern California Bay Area residents who participated in a research study focused on well‐being are not representative of the general population. Overall, the Bay Area has significant gaps in socioeconomic status among residents (Kawachi, 2002; Parise & Caggiano, 2017). As a result of the increasing advancement of the technology industry, some of the wealthiest citizens in the United States live in the Bay Area (i.e., Silicon Valley) (Stephens et al., 2019). Thus, many of the participants who completed the surveys may have had access to extensive financial and health resources, resulting in additional time to pursue health‐promoting behaviors. Likewise, we observed that the subset of WELL participants who completed both the WELL Diet survey and the FFQ were older, more educated, likely to be married, and more physically active than non‐FFQ completers. Also, the relatively high demographic homogeneity among our sample of FFQ completers may have led to a limited range of dietary differences that may in turn have underestimated the strength of the true correlation of the two assessment tools. Finally, we recognize the small proportion of participants who opted to complete the FFQ (~300 out of ~4,000) as a limitation.

Our findings add to the accumulating evidence base that suggests brief diet quality assessments may play a strategically important role in the methodological advancement of diet‐related studies. This bears, particularly, on those assessments where diet quality is only one of many variables being assessed and where respondent burden and cost are of concern. Further studies are necessary to support the generalizability of the WELL Diet Score in assessing diet quality in other populations and research settings, and its usefulness in assessing changes in dietary behaviors related to changes in wellness outcomes. Overall, this study serves as a foundational step toward applying more practical and useful nutrition assessment methods in the WELL study.

## CONFLICT OF INTERESTS

The authors declare that they do not have any conflict of interest.

## ETHICAL STATEMENTS

This study conforms to the Declaration of Helsinki and U.S. Medicines Agency Guidelines for human subjects. This study's protocols and procedures were ethically reviewed and approved by the Institutional Review Board of Stanford University. Informed consent was obtained and documented for all of WELL participants, and a statement confirming informed consent was obtained. Human and animal testing was unnecessary in this study.

## Supporting information

Appendix S1‐S3
