# Supplementary material for: The WELL diet score correlates with the alternative healthy eating index‐2010
Source: Food Sci Nutr. 2020 May 6;8(6):2710–8. doi: 10.1002/fsn3.1558 (PMC7300046; doi:10.1002/fsn3.1558)
Supplement: Supplementary file 1 — Appendix S1‐S3 [file FSN3-8-2710-s001.docx]

**APPENDICES**

**Appendix 1:** WELL Diet Scoring Methodology

| **WELL Diet Questions** | **Scoring Valence** | **Criteria for min score (0)** | **Criteria for max score (10)** | **WELL Diet Scoring** | | | | | | | | | |
| --- | --- | --- | --- | --- | --- | --- | --- | --- | --- | --- | --- | --- | --- |
|  |  |  |  | **Never** | **1x month** | **2-3 x month** | **1-2 x week** | **3-4 x week** | **5-6x week** | **1 x day** | **2-3 x day** | **4-5 x day** | **≥6 day** |
| How often did you eat **vegetables**? | positive | ≤1 x month | ≥4 x day | 0 | 0 | 1 | 2 | 4 | 6 | 8 | 9 | 10 | 10 |
| How often did you eat **fruit**? Include fresh, frozen, or canned fruit. Do not include fruit juice. | positive | ≤1 x month | ≥2 x day | 0 | 0 | 1 | 2 | 4 | 6 | 8 | 10 | 10 | 10 |
| How often did you eat **whole grains** and **whole grain** **products**, such as whole grain bread or muffins, whole grain rice (i.e., brown rice), bulgur, whole grain pasta, or whole grain cereal? | positive | ≤1 x month | 2-5 x day | 0 | 0 | 1 | 2 | 4 | 6 | 8 | 10 | 10 | 8 |
| How often did you drink **sugar sweetened beverages or 100% fruit juice**? Include soda, sweetened energy drinks, sweetened fruit drinks, or coffee/tea that you add sweetener to. Do not include diet drinks. | negative | ≥1 x day | 0 | 10 | 9 | 8 | 6 | 4 | 1 | 0 | 0 | 0 | 0 |
| How often did you eat **sugar-sweetened baked goods or candy**, such as cookies, donuts, pastry, and candy bars? | negative | ≥1 x day | 0 | 10 | 9 | 8 | 6 | 4 | 1 | 0 | 0 | 0 | 0 |
| How often did you eat cooked or canned **beans or lentils**? Include refried, baked, black, or garbanzo beans, beans in soup, soybeans, edamame, tofu or lentils. Do not include long green beans (long green beans should be counted as vegetables). | positive | 0 | ≥2 x day | 0 | 1 | 2 | 4 | 6 | 8 | 9 | 10 | 10 | 10 |
| How often did you eat **nuts, seeds, or nut butter**, such as peanut butter or almonds? | positive | 0 | 1-3 x day | 0 | 1 | 2 | 4 | 6 | 8 | 10 | 10 | 8 | 6 |
| How often did you eat **red meat or processed meat**, such as bacon, sausage, bologna, ground beef, steak, beef ribs, roast beef, or hamburger sandwich? | negative | ≥2 x day | ≤1 x month | 10 | 10 | 8 | 6 | 4 | 2 | 1 | 0 | 0 | 0 |
| How often did you eat **fish**? | positive | 0 | ≥1 x week | 0 | 4 | 7 | 10 | 10 | 10 | 10 | 10 | 10 | 10 |
| How often did you eat **high sodium processed foods** like canned soup, pizza, prepared pasta dishes, and savory snacks (chips, popcorn, pretzels)? | negative | ≥2 x day | 0 | 10 | 9 | 8 | 6 | 4 | 2 | 1 | 0 | 0 | 0 |
| How often did you eat **fast food**? Include fast food eaten at work, at home, or at fast-food restaurants, carryout or drive through. Include food you get at places like McDonald's, KFC, Panda Express, Taco Bell, or other fast food restaurants. | negative | ≥3 x week | 0 | 10 | 8 | 5 | 2 | 0 | 0 | 0 | 0 | 0 | 0 |
| How often did you **prepare your own meal** (cook food)? | positive | 0 | ≥2 x day | 0 | 1 | 1 | 2 | 4 | 6 | 8 | 10 | 10 | 10 |
|  |  |  |  |  |  |  |  |  | **Total Possible Points:** | | | | **120** |

**Appendix 2:** Distribution of the a) WELL Diet Score and the b) AHEI-2010 (N=248)

| **a)** | 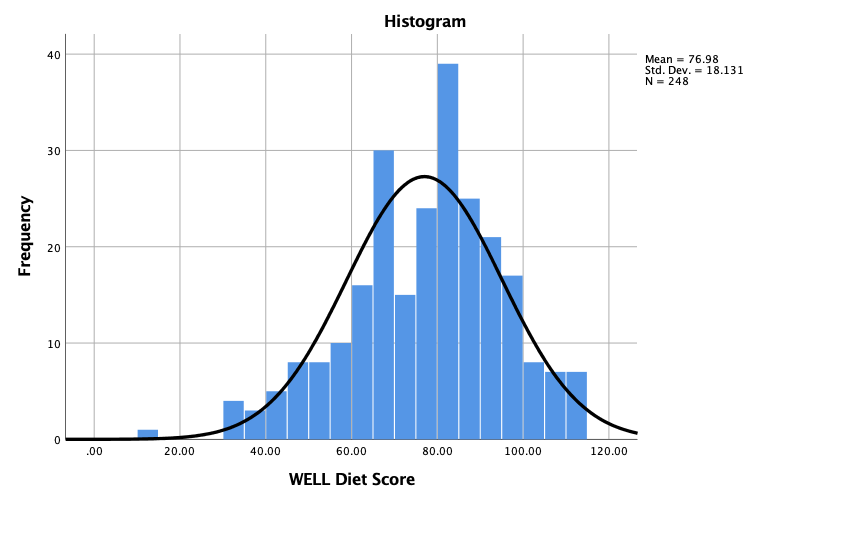 | **b)** | 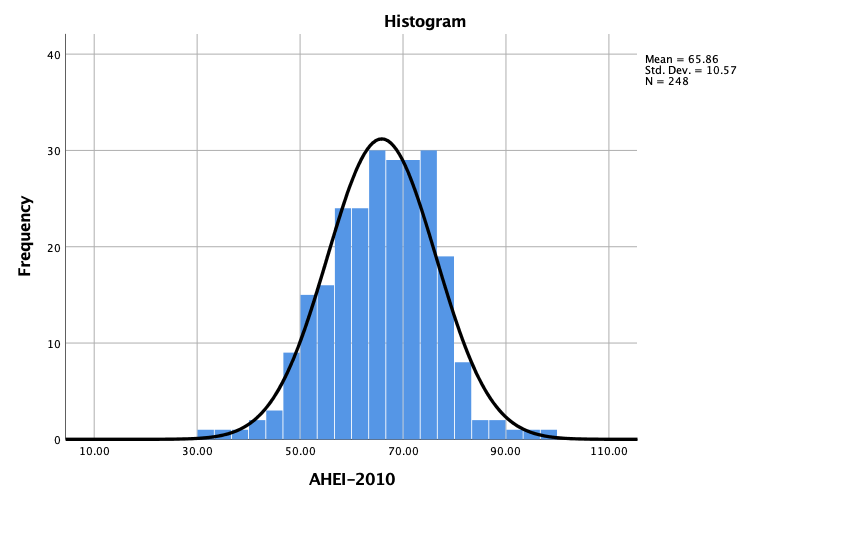 |
| --- | --- | --- | --- |

**Appendix 3:** Univariate linear regression between the WELL Diet Score, AHEI-2010, sociodemographic determinants of diet quality and related health factors (N=248)

| **Characteristics** | **WELL Diet Score**  **β (SE)** | **p-value** | **AHEI-2010**  **β (SE)** | **p-value** |
| --- | --- | --- | --- | --- |
| **Age, years** | 0.2 (0.06)*** | ≤0.001 | 0.2 (0.04) ** | 0.003 |
| †**Level of Education** | 0.2 (1.43) *** | ≤0.001 | 0.2 (0.83) *** | ≤0.001 |
| ‡**BMI, kg/m2** | -0.2 (0.19) *** | ≤0.001 | -0.1 (0.01) | 0.06 |
| **Current smoker** | 0.01 (0.82) | 0.86 | 0.02 (0.48) | 0.76 |
| §**Physically Active** | 0.3 (2.23) *** | ≤0.001 | 0.2 (0.13) ** | 0.002 |

***p-value ≤0.001, ** p-value ≤0.01, p-value ≤0.05

†Education, missing n=26

‡BMI, missing n=18

§Physical activity, missing n=3
